# VPPIPP and IPPVPP: Two Hexapeptides Innovated to Exert Antihypertensive Activity

**DOI:** 10.1371/journal.pone.0062384

**Published:** 2013-04-30

**Authors:** Fengyun Ding, Bingjun Qian, Xin Zhao, Shanqi Shen, Yun Deng, Danfeng Wang, Feng Zhang, Zhongquan Sui, Pu Jing

**Affiliations:** 1 Department of Food Science and Engineering, School of Agriculture and Biology, Shanghai Jiao Tong University, Shanghai, China; 2 Yancheng Institute of Health Sciences, Jiangsu, China; 3 Bor Luh Food Safety Center, Shanghai Jiao Tong University, Shanghai, China; Universidade Federal do Rio de Janeiro, Brazil

## Abstract

In this study, two hexapeptides of IPPVPP and VPPIPP were innovated by using two commercial antihypertensive peptides IPP and VPP as two domains *cis*-linked and *trans*-linked, respectively. The IPPVPP and VPPIPP were chemically synthesized and evaluated for the antihypertensive activity *in vitro/vivo*. The *in vitro* ACE-inhibitory study showed that VPPIPP (34.71±4.38%) has a significantly stronger activity than that of IPPVPP (13.17±0.25%) at a treatment concentration of 10 µmol/L, but it was weaker than the commercial IPP (56.97±2.40%) (*P*<0.05). However, VPPIPP, IPPVPP, and IPP lowered the systolic blood pressure by 21±0.9%, 17.4±1.3% and 17.5±0.9%, respectively, in rats at 1.5 mg/kg body weight dosage. The result was consistent with the mRNA level of sarcoplasmic reticulum Ca^2+^, Mg^2+^ -ATPase Gene (*SERCA 2a*) in rat hearts. Additionally, VPPIPP and IPPVPP showed no negative impact on blood glycometabolism. The results suggested that the two hexapeptides could be potent bioactive peptides in functional foods for people with high blood pressure.

## Introduction

Hypertension is a world-wide health issue encountered by different ages of people and becomes one of the most important preventable causes of premature morbidity and mortality. According to the World Health Organization's 2012 *World Health Statistics*, the population with hypertension has drastically increased from 600 million in 1980 to nearly 1 billion in 2008 in both developed and developing countries. One in three adults worldwide and half of adults in some Africa countries have high blood pressure, and it is estimated to cause 7.5 million deaths, about 12.8% of all annual deaths worldwide [1]. Not surprisingly, it has gained top public and scientific concern due to its prevalence and risk of cardiovascular disease and death.

Studies on the mechanism of the hypertension occurrence revealed that the angiotensin converting enzyme (ACE), a component of the renin–angiotensin system (RAS), catalyzes the formation of the strong vasoconstrictor angiotensin II (Ang II) from angiotensin I (Ang I), contributing to the maintenance of normal blood pressure [2]. Therefore, ACE has become the major target of medicine in the clinical treatment of hypertension, and some ACE-inhibitors were developed, such as captopril, enalapril, lisinopril, tenormin, and temocapril. However, with the prolonged usage, they might produce side-effects to some patients, causing dry-cough, headache, dizziness, itching and rash [3].

In the past decades, researchers have focused on the natural nutraceutical resources and investigated the potential antihypertensive activity *in vitro* by ACE-inhibitory activity [4], [5]. Some natural phytochemicals from plants, including rutin, quercetin glycoside, epicatechin-tetramer and cyanidin-3-O-sambubioside have been extensively studied as a substitute for chemo-pharmaceuticals [6]–[8]. Also, some antihypertensive peptides have been identified from hydrolyzed or fermented milk and other dietary sources [9]. Among them, the Isoleucine-Proline-Proline (IPP) and Valine-Proline-Proline (VPP) with IC50 of 5 µmol/L and 9 µmol/L were the typical ones. They are generally recognized as safe (GRAS) by the U.S. Food and Drug Administration (FDA) and have been approved for use in foods to help reduce blood vessel constriction [10].

Most ACE-inhibitory peptides contain 3 to 9 amino acid residues, whereas the good antihypertensive peptides from milk have 6 to 10 residues. It is shown that the peptide structure properties including C-terminal amino acid sequence have critical impact on the ACE-inhibitory activity in previous research. The C-terminal of the most natural ACE-inhibitory peptides is Ala-Pro or Pro-Pro residues [11]–[19], and the high ACE-inhibitory activity peptides derived from tuna myoprotein [11], cheese protein [15], and wheat protein [19] all have the Pro residue C-terminal, indicating that the ACE-inhibitory peptides with Pro in the C-terminal position appeared with a great activity. Additionally, the ACE-inhibitory peptides from casein always have hydrophobic amino acid residues at each of the three C-terminal positions, suggesting that the hydrophobicity is another factor important for the ACE-inhibitory peptide activity. Besides, peptides with aromatic or alkaline amino acids at the N-terminal showed an enhanced antihypertensive activity [15]–[19].

Previous studies demonstrated that the transport of intact peptides including oligopeptides (i.e. dipeptide, tripeptide, and hexapeptide) [10], [20], and polypeptides (i.e. insulin) [21], from the intestinal lumen into blood is a unique phenomenon, differing from the regular digestion and absorption of food. Additionally, dipeptides, tripeptides and hexapeptides absorbed intactly into blood circulation *in vivo*, retained their biological activity [10], [20].

In this study, we innovated two hexapeptides (Isoleucine-Proline-Proline-Valine- Proline-Proline, IPPVPP; Valine-Proline-Proline-Isoleucine-Proline-Proline, VPPIPP) based on the previous studies on the preferable structure of antihypertensive peptides. The newly-synthesized peptides were evaluated for their antihypertensive activities *in vitro* and *in vivo*.

## Materials and Methods

### Materials and chemicals

The tripeptides (IPP and VPP) and hexapeptides (IPPVPP and VPPIPP) used in this study were chemically synthesized by GL Biochem (Shanghai) Ltd (Shanghai, China) and their purity was more than 95%. The spontaneously hypertensive, strain SHR/Slac male rats aged 20 weeks were purchased from Shanghai Slac Laboratory Animal Co. Ltd. (Shanghai, China). Chemical reagents were from Sigma Chemical Co. (Louis, USA) and Sinopharm Chemical Reagent Co. Ltd. (Shanghai, China). Hippuryl-l-histidyl-l-leucine (HHL) and Hippuric acid (HA) were obtained from Sigma Chemical Co. (Louis, USA).

### Preparation of ACE from rabbit lungs and determination of enzyme activity

ACE extracts were prepared and its activity was determined according to the method of Nakamura et al. [22] and Wu & Ding [23] with some modifications. Briefly, the fresh rabbit lung without connective tissues was cut into pieces and washed with precooled NaCl (0.7%) to get rid of blood. The tissue pieces were homogenized in precooled 5-fold the volume of fresh rabbit lung weight (v/w, mL/g) of 100 mM sodium borate buffer (BBS) (pH 8.3). The mixtures were then filtrated with 3-ply gauze followed by ultracentrifuged for 40 min (40,000 *g*, at 4°C). The supernatant as crude ACE extracts was retained and stored at 5°C [24].

The ACE activity was assayed using the method described by Wu & Ding [23]. It was based on the hydrolysis of hippuryl-L-histidyl-L-leucine (HHL) by ACE to hippuric acid (HA) and histidyl-leucine (HL) as products. The HA release from HHL was directly related to the ACE activity. One unit (U) of ACE was defined as the amount of enzyme catalyzing the release of 1 µmol/L HA from the substrate HHL per minute at 37°C. Briefly, 30 µL of ACE crude extracts were mixed with 100 µL of BBS (pH 8.3). The mixture was incubated at 37°C for 10 min. Then, 200 µL of 5 mmol/L HHL solution substrate were added and the reaction mixture was incubated at 37°C for 20 min. The reaction was stopped by the addition of 250 µL of 1 mol/L HCl. 10 µL of solution were analyzed by reversed-phase HPLC and detected at the wavelength of λ = 228 nm. The column was eluted with 20% acetonitrile (in water, v/v) containing 0.1% (v/v) TFA on AKATA purifier 10 with a flow rate of 1.0 mL/min. The blank experiment was executed with premixing 250 µL of 1 mol/L HCl and 30 µL of ACE crude extracts to inhibit the activity of ACE.

### Determination of ACE inhibitory activity of the selected tripeptides *in vitro*

Evaluation of ACE inhibitory activity was assayed using the same method described in above section with some modifications. Briefly, 2 µL of ACE crude extracts were mixed with 80 µL of 10 µmol/L each selected peptide solution. The mixture was incubated at 37°C for 10 min, after which 200 µL of 5 mmol/L HHL solution substrate were added and the reaction mixture was incubated at 37°C for 20 min. Finally, 250 µL of 1 mol/L HCl were added to stop the reaction and 10 µL of solution were used to analyze HA release from HHL by reversed-phase HPLC with the same condition described above and detected at the wavelength of λ = 228 nm. Controls with equal volume of BBS as sample substitute were included for each measurement, and the blank experiment was executed with premixing 250 µL of 1 mol/L HCl and 2 µL of ACE crude extracts to inhibit the activity of ACE, firstly.

The evaluation of ACE inhibition was based on the measurement of the HA peak areas, and the degree of ACE inhibition (%) was calculated according to Eq.1:
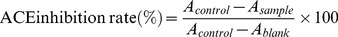
(Eq.1)


Where *A_control_* was the HA peak area of the buffer instead of tested samples), *A_blank_* was the HA peak area of the reaction blank when HCl was added before HHL, and *A_sample_* was the HA peak area of tested samples.

### Ethics statement

All animal studies were carried out in strict accordance with the recommendations in the Guide for the Care and Use of Laboratory Animals of the Shanghai Jiao Tong University School of Pharmacy. The protocol was approved by Shanghai Municipal Science and Technology Commission, the Shanghai Municipal Laboratory Animal Management Office (Permit Number: 11ZR1416200), and the Committee on the Ethics of Animal Experiments of the Shanghai Jiao Tong University School of Agriculture and Biology (Permit Number: NQN201001).

### Assay of blood pressure of SHR *in vivo*

The spontaneously hypertensive, strain SHR/Slac male rats (Production Permit Number: SCXK (Shanghai) 2007-0005; Body weight, average ∼400 g; Age, 20 weeks old), were purchased from Shanghai SLAC Laboratory Animal Co. Ltd. (Shanghai, China). The SHR were housed in cages and maintained under specific pathogen-free conditions on a cycle of 12 h of light and 12 h of darkness at Center of Experimental Animal, School of Pharmacy, Shanghai Jiao Tong University (Use Permit Number: SYXK (Shanghai) 2007-0025). The temperature and humidity in the cages were controlled at 24±1°C and 60±5%, respectively. SHRs were acclimatized for 7 days prior to experimentation. The rats were fed a standard laboratory diet (SLACOM; Shanghai, China), and tap water was freely available. After the hexapeptide water-solution (dose, 1.5 mg/kg body weight) injection with intragastric administration in six SHR, the systolic blood pressure (SBP) of each rat was measured by the tail-cuff method with a programmable electrosphygmomanometer (model SoftronBP-98A; Softron Beijing Incorporated, Beijing, China) at 1 h interval. A heater lamp was on the tail to improve signal quality. To obtain an accurate SBP reading, rats were first habituated to the SBP measurement device. Definitive measurements began when rats remained unperturbed into the chamber throughout the inflation-deflation cycles. The SBP in each SHR with administration of distilled water (2 mL) was used as the control value. The data are expressed as means and standard errors (n = 6).

To evaluate whether the lowering SBP effect was the dose-dependence relative to the test peptides, the SHR were also treated with 0.75 mg/kg BW peptides water-solution as above program. And the relative lowering SBP effect was calculated as the following equation (Eq.2):
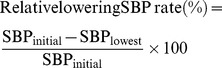
(Eq.2)where SBP_initial_ is the initial SBP values of SHR right before the peptide administration, SBP_lowest_ is the lowest SBP value obtained within 5 h after the administration with the tested peptides.

### Quantitative real time-PCR analysis of *SERCA 2a* gene

Total RNA was isolated from heart peeled from the SHR 3 h after fed with the tripeptide IPP, hexapeptides IPPVPP, or VPPIPP using Trizol reagent (Generay) as instructed by the supplier. After treatment with DNase (Promega), 0.3 mg RNA was employed to synthesize the oligo (dT) primed first-strand cDNA using the ReverTra Ace-a-First Strand cDNA synthesis kit (TOYOBO). Quantitative real time-PCR (qRT-PCR) analysis was performed using SYBR Premix EX Taq (TaKaRa) on a Rotor-Gene RG3000A detection system (Corbett Research) as described by Zhang et al. (2010) with the exception of the annealing temperature at 58 °C when using the primers SERCAF (5′-TCTGACTTTCGTTGGCTGTG-3′) and SERCAR (5′-GCCTTTGTTATCCCCAGTGA-3′) [25], [26]. Gene GAPDH was used as reference gene with primer GAPDHF (5′-CCATGGAGAAGGCTGGG-3′) and GAPDHR (5′-CAAAGTTGTCATGGATGACC-3′) [26]. Three biological replicates were used, and each with three technical repeats.

### Effect of tripeptides on blood glucose level *in vivo*

To evaluate the effect of the selected tripeptides on glycometablism after administration *in vivo*, the SHRs were tail bled 3.5 h after administration with the selected tripeptides at a dose of 1.5 mg/kg BW, and blood glucose level was determined by One Touch Ultra test strips using the One Touch Ultra blood Glucose monitoring system (LifeScan, Inc. Milpitas, USA).

### Statistical analysis

Three replicate trials for each experiment were performed. Analysis of variance was performed and means separated using software SPSS version 14.0. Significant difference (*P*<0.05) between means were identified using least significant difference (LSD) procedures.

## Results and Discussion

### ACE-inhibitory activity assay of selected hexapeptides *in vitro*

To evaluate the antihypertensive activity of the innovated hexapeptides, the two hexapeptides (IPPVPP and VPPIPP) and the commercialized tripeptides (IPP and VPP as positive controls) were chemically synthesized to test its ACE inhibitory activity *in vitro*. The VPPIPP and IPPVPP showed different ACE inhibitory activity comparing to the commercial IPP and VPP at the treatment concentration of 10 µmol/L (Table 1), following an order: IPP (56.97%)>VPP (37.62%)≈VPPIPP (34.71%)>IPPVPP (13.17%) at the 0.05 level by least significant difference test (Tukey HSD). In our test system, the ACE inhibitory activity of IPP and VPP at 10 µmol/L, used as positive controls, was a little weaker than that reported by Nakamura et al. [22]. The ACE-inhibitory activity of IPP (56.97%) was significantly stronger than that of VPP (37.62%) as two positive controls (*P*<0.05), indicating that the test system worked well. However, the values of IPP and VPP were slightly weaker than those reported by Nakamura et al. [22], which might attribute to the differences in detection assays, substrates, test conditions, or/and ACE origins [27].

**Table 1:** ACE Inhibitory Activity of the Screened Peptides *in vitro*.

Peptides	ACE inhibition rate (%)
IPP	56.97±2.40^a^
VPP	37.62±6.68^b^
IPPVPP	13.17±0.25^c^
VPPIPP	34.71±4.38^b^

ACE inhibition rate (%) was expressed as mean ± SD. Values with the same letters are not significantly different (*P*<0.05) using LSD.

Previous studies described that hydrophobic amino acids at each of the three C-terminal positions of antihypertensive peptide candidates were favorable, largely because Pro at the ultimate C-terminal position could gave them resistance to digestion in gastrointestinal tract and also improve exceptionally the affinity to ACE [28], [29]. Therefore, the antihypertensive activity of the two hexapeptides was further analyzed *in vivo* using SHR rats.

### Effect of IIPVPP and VPPIPP on blood pressure in SHR

**Figure 1:**
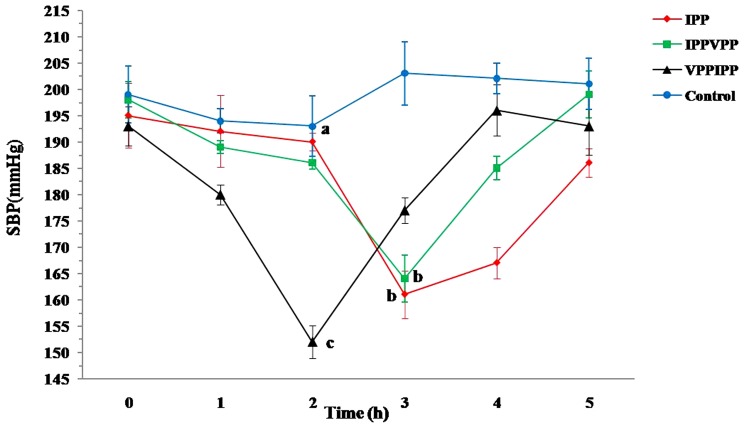
Lowering SBP effect of single oral administration of IPPVPP and VPPIPP on SHR spontaneously hypertensive rats. IPPVPP, VPPIPP, and IPP (the positive control) were fed to the three groups of SHR with at a dose of 1.5 mg/kg BW, respectively; the distilled water as the control was fed to a SHR group at the same dosage. The SBP was expressed as mean ± SD. Points with the same letters mean no significant difference (*P*<0.05).

To investigate the blood pressure lowering effect of the hexapeptides (IPPVPP and VPPIPP), male SHRs were administrated intragastrically with the hexapeptide water-solutions. The SBP of SHRs had been all decreased significantly within 2–3 hrs after administration (*P*<0.05) (Figure 1). The SBP recovered to the initial state5 h after administration. It showed that the antihypertensive effect of these products was transient. The lowest SBP values were obtained at the time of 2, 2, 3, and 3 h for the control, VPPIPP, IPPVPP, and IPP, respectively. The result showed that VPPIPP decreased the SBP from 193±3.7 mmHg as low as to 152±3.1 mmHg, exhibiting the strongest antihypertensive activity (*P*<0.05). IPPVPP (SBP_lowest_ = 164±4.4 mmHg) showed no significant difference from the IPP (SBP_lowest_ = 161±3.1 mmHg). The IPP reduced SBP from 195±6.2 mmHg to 161±3.1 mmHg (1.5 mg/kg BW dosage), although less effective than results reported by Nakamura et al. (1995) that IPP decreased the SBP of SHR by 21.7±4.1 mmHg (0.3 mg/kg BW dosage) in SHR [30]. In the present study, VPPIPP that decreased SBP by ∼41 mmHg (dose: 1.5 mg/kg BW) in SHR, was comparable or even better than antihypertensive peptides reported in previous studies [31]–[33]. Maeno et al. (1996) reported that administration of 2 mg/kg BW of heptapeptide KVLPVPQ (β-casein 169–175aa) resulted in a maximal decrease in SBP by 31.5±5.6 mmHg in SHR [31]. Abukabar et al. (1998) demonstrated that proteinase K hydrolysate of whey had the ability to reduce SBP by 55±2.6 mmHg, following an administration of 8 mg/kg BW in SHR [32]. Nurminen et al. (2000) showed that the whey protein derived tetrapeptide YGLF could reduce SBP by 23±4 mmHg following subcutaneous administration of 0.1 mg/kg BW in SHR [33]. The decrease in the SBP observed for captopril (antihypertensive positive control, 50 mg/kg) was about 53±4 mmHg in male SHR 4–6 h after administration [34]. Those suggest that VPPIPP was potent to be commercialized as antihypertensive peptide in future.

Apparently the VPPIPP even showed a significantly greater effect on the lowering SBP than the commercialized IPP *in vivo* (*P*<0.05), and it was not consistent to the result of ACE-inhibitory activity *in vitro*, which might attribute to the hydrolysis of VPPIPP by peptidase in intestinal tract or its instability in the blood or other mechanisms [27]. We also proposed that the VPPIPP hydrolysate might contain the active fragment such as IPP, which was already proved to be one of the strongest antihypertensive potential peptide [10]. Secondly, the C-domain catalytic sites of the somatic form of ACE are the susceptible bonds around Pro, Phe and His of Ang I (DRVYIHPFHL) [27], whereas VPPIPP has a good chance to generate the hydrolysate with a Pro at the fourth position started from the carboxyl terminal end, structurally similar to Ang I. That might be a competitive factor additionally contributing to the lowering SBP effect of VPPIPP. Those inferences need to be confirmed in future study. Additionally, it was noticed that the lowest SBP in our study was reached 1 h earlier than that the report by Nakamura et al., possibly due to the different forms of fed samples. In the study we applied a water solution, whereas Nakamura et al. used a viscous aqueous sodium caseinate solution that might result in a delayed absorption [30].

**Figure 2:**
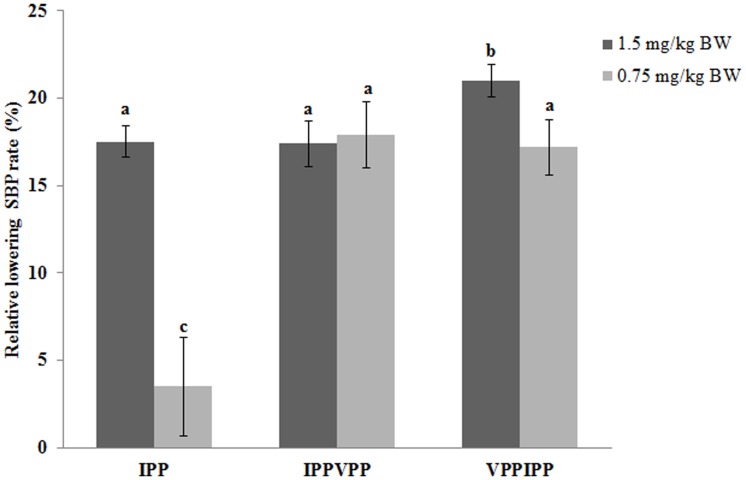
Dose effect of IPPVPP and VPPIPP on SHR spontaneously hypertensive rats. IPPVPP, VPPIPP, and IPP (the positive control) were fed to the three groups of SHR with at a dose of 0.75 or 1.5 mg/kg BW, respectively. Column with the same lowercase are not significant different (*P*<0.05) using LSD.

To better differentiate the lowering SBP effect of tested peptides and understand the dose-effect relationship, the relative lowering SBP rate (%, equation described in the section of materials and methods) was generated to illustrate the effect of tested samples on the lowering SBP. A higher value of relative lowering SBP indicates a greater activity. Figure 2 showed that VPPIPP still had a significantly greater effect than others did at 1.5 mg/kg BW dosage (*P*>0.05). However, VPPIPP did not show significantly different from IPPVPP and IPP at 0.75 mg/kg BW dosage. Additionally, both VPPIPP and IPP showed a dose-dependent effect on lowering rat SBP (*P*>0.05) whereas IPPVPP did not. However, additional work needs to be done on a lower effective dosage(<0.75 mg/kg BW) of the tested hexapeptides in future.

### Transcriptional regulation on *SERCA 2a* gene of hexapeptides

**Figure 3:**
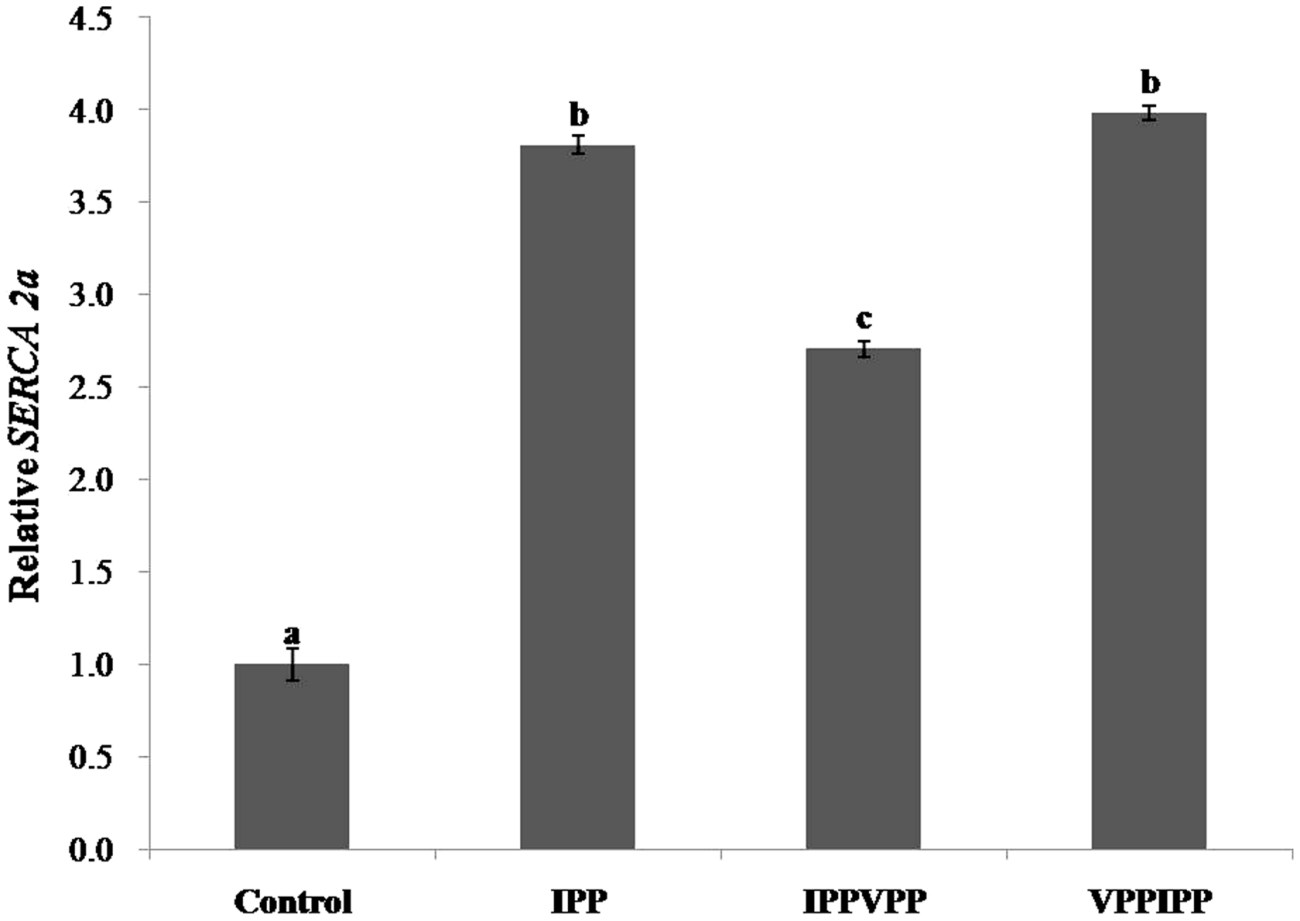
Quantitative PCR analysis of mRNA level of *SERCA 2a* gene. Error bars indicate SD; each reaction has three quantitative PCR biological replicates. Column with the same lowercase letters are not significantly different (*P*<0.05) using LSD.

To further evaluate the antihypertensive function of the tested peptides, mRNA level of *SERCA 2a* gene in SHRs heart tissue was analyzed by Real-time PCR. In Figure 3, the transcriptional levels of *SERCA 2a* gene for VPPIPP and IPPVPP groups were 3.98 and 2.70 times of the control, respectively (*P*<0.05). The VPPIPP groups showed no difference from IPP group (3.80 times of the control) in the transcriptional level of *SERCA 2a* gene, but significantly greater than IPPVPP (*P*<0.05). In the myocardial tissue, Ang II receptor (AT1) is the key element for the cardiocytes to accept stimulation from Ang II and to induce signal transduction which could regulate some corresponding gene expression level [35]. Cui et al. [26] reported that Ang II infused could down-regulate transcriptional level of *SERCA 2a* gene in rat models of cardiac hypertrophy, which was mediated by AT1. In the renin-angiotensin system, ACE can convert Ang I to the potent vasoconstrictor, Ang II, to increase blood pressure [2]. Based on those previous reports and our results, we proposed that VPPIPP, IPPVPP and IPP might competitively inhibit ACE from catalyzing Ang I into Ang II *in vivo*. Consequently, the reduction of Ang II resulted in lowering blood pressure and the up-regulation of the mRNA level of *SERCA 2a* gene, which were consistently confirmed in this study (Figures 1, 2, and 3). SERCA could increase intracellular Ca2+ uptake to mediate smooth and cardiac muscle relaxtion, and thereby regulates blood pressure [36]. Besides increasing arterial pressure, Ang II mediated by the plasma membrane AT1 can increase sodium and fluid retention and enhance sympathetic adrenergic function, which are also related with blood pressure [37]. However, all results suggested that VPPIPP could be further applied as nutriceutical ingredients in functional food to prevent the development of hypertension.

### Effect of glycometabolism assay of tested hexapeptides *in vivo*

It is necessary to evaluate the ACE-inhibitor effect on the blood glucose level of hyperpietics simultaneously since blood sugar was positively correlated with blood pressure (systolic and diastolic), and this correlation was independent of common associations with age, body mass index (BMI) and heart rate [38]. Table 2 showed that all tested peptides and control had no significant difference 3.5 h after administration (*P*<0.05), suggesting that the designed hexapeptides had no negative effect on glycometabolism, just as same as captopril that has been proved by McGrowder et al. [39] to have no significant effect on postprandial blood glucose levels in the normoglycemic and normotensive animal model. The VPPIPP and IPPVPP are potent antihypertensive nutriceuticals.

**Table 2:** Analysis of Effect on Blood Glucose Level of SHR Fed with IPPVPP and VPPIPP.

SHR groups	Blood Glucose Concentration (mmol/L)
IPP	3.55±0.45^a^
IPPVPP	4.20±0.60^a^
VPPIPP	4.30±0.15^a^
Control	4.00±0.30^a^

IPP,IPPVPP and VPPIPP represented the SHR groups fed with peptides of IPP, IPPVPP and VPPIPP, respectively, at dose of 1.5 mg/kg BW; control group was fed with distilled water. Blood glucose level was expressed as mean ± SD. Values with the same letters are not significantly different (*P*<0.05) using LSD.
